# Working in rural areas – the experiences of Umthombo Youth Development Foundation graduates

**DOI:** 10.4102/phcfm.v6i1.673

**Published:** 2014-12-03

**Authors:** Andrew J. Ross

**Affiliations:** 1 Department of Family Medicine, University of KwaZulu-Natal, South Africa

## Abstract

**Background:**

Recruiting and retaining healthcare professionals (HCPs) for rural areas is challenging throughout the world. Although rural origin HCPs have been identified as being the most likely to work in rural areas, only a small number of rural-origin South African scholars are trained as HCPs each year and many do not return to work in rural areas.

**Aim:**

The aim of this article was to present the experiences of rural-origin HCPs who returned to work in a rural area after graduation.

**Setting:**

Umthombo Youth Development Foundation has been running an innovating rurally-based scholarship scheme since 1999. By December 2013, 184 students supported by the scheme had graduated and all had returned to work in a rural area for a period of time.

**Methods:**

This was a qualitative study using a life history methodology to explore the educational experience of six rural-origin HCPs working in rural areas.

**Results:**

The four themes that emerged from the data were: (1) contribution to service delivery; (2) professional development (3) the challenges and frustrations of working in rural hospitals; and (4) the impact of working as an HCP.

**Conclusion:**

Rural-origin HCPs are willing to return and work in rural areas. However, context and content factors need to be addressed if a work-back scholarship scheme is to be a long-term strategy for the recruitment and retention of HCPs.

## Abstract (fr)

**Travail dans les zones rurales – les expériences des Diplômés de la Fondation Uthombo pour le Développement de la Jeunesse.**

**Contexte:**

Le recrutement et la rétention des Professionnels de la Santé (HCP) pour les zones rurales est un problème mondial. Bien qu’on considère que les HCP d’origine rurale seront probablement ceux qui accepteront de travailler dans les régions rurales, un très petit nombre d’étudiants sud-africains d’origine rurale reçoivent une formation de HCP chaque année et beaucoup d’entre eux ne retournent pas travailler dans les zones rurales.

**Objectif:**

Cette étude a pour objectif de décrire les expériences des HCP d’origine rurale qui sont retournés travailler dans les régions rurales après leurs études.

**Cadre:**

la Fondation Umthombo du Développement de la Jeunesse dirige un système de bourses innovant en région rurale depuis 1999. En décembre 2013, 184 étudiants boursiers avaient reçu leur diplôme et tous étaient retournés travailler un certain temps dans une zone rurale.

**Méthode:**

Cette étude qualitative a utilisé la méthode de l’histoire de vie pour étudier ’expérience éducationnelle de six HCP d’origine rurale travaillant dans les zones rurales.

**Résultats:**

Trois thèmes ont émergé des données: (1) la contribution à la prestation de services; (2) Les défis et frustrations de travailler dans des hôpitaux ruraux; et (3) l’impact de travailler comme HCP.

**Conclusion:**

Les HCP d’origine rurale veulent bien retourner travailler en zone rurale. Cependant, il faudra examiner le contexte et le contenu si l’on veut que le système de bourses à rembourser par le travail devienne une stratégie de recrutement et de rétention à long terme des HCP.

## Introduction

Without sufficient numbers of well-trained professional staff, key health outcomes will never be realised.^1^ Many rural areas in South Africa have a high burden of infectious diseases, high under-five mortality and reduced life expectancy at birth. The 10 districts with the highest deprivation index in South Africa in 2008 were all rural.^2^ There are major inequalities between staffing levels at hospitals in rural and urban areas which contribute to poor health outcomes.^2,3^ These disparities remain, despite the commitment of the National Department of Health to ‘Health for All’^4^ and the prioritisation of recruitment of healthcare professionals (HCPs) for rural areas.^5^ Maternal, child and infant mortality rates increase as the number of healthcare workers (HCWs) decreases, whilst increasing the number of HCWs has been shown to improve health outcomes in underserved areas.^6^

Rural-origin healthcare professionals (HCPs) have been identified as being the most likely to work in rural areas after qualification and to contribute to improving health outcomes in these areas.^7^ However, only a small number of rural-origin South African scholars are trained each year as HCPs^8^ and finding staff for rural hospitals is an ongoing challenge. Absent role models, dysfunctional families, poorly-performing schools and limited financial support all make it challenging for rural-origin students to gain access to tertiary institutions in order to train as HCPs.^9,10,11,12,13,14^ Compounding the issue of staffing for rural institutions is the fact that only a small percentage of rural-origin HCPs actually choose to work in rural institutions.^15^

The Friends of Mosvold scholarship scheme (now the Umthombo Youth Development Foundation Scholarship Scheme [UYDF SS]), an innovative rural scholarship scheme, was started in 1999. It was based on international research which showed that the training of rural scholars was potentially a long-term solution to the chronic staff shortages in rural and remote areas. Although there are a number of definitions of ‘rural’ (based on distance from urban areas, resources available, geographic location, economic activity, etc,),^16^ by any definition students supported by UYDF SS would be considered to be of rural origin. By November 2013, 184 HCPs supported by the scheme had graduated. All graduates have returned to work at a rural district hospital near where they live to fulfil their work-back obligation. Less than 10% of graduates have bought themselves out of a portion of their work-back obligation and more than 60% have continued to work in a rural area after their work-back obligations were complete.^17^

The willingness of these graduates to return and work in rural hospitals stimulated this research project which is part of a PhD dissertation looking at the educational journeys of rural-origin healthcare professionals working in rural areas. The aim of this article was to present the experiences of these rural-origin HCPs who returned to work in rural areas after graduation. It is hoped that this article will contribute to the discussion regarding the selection and support of rural-origin scholars and strategies for the staffing of rural health care institutions.

## Research methods and design

### Study design

This was a qualitative study using a life history methodology to explore the educational experience of rural-origin HCPs.

### Selection of participants

Six rural origin HCPs were selected purposively from UYDF SS graduates. The criteria for selection of graduates were: (1) their willingness to participate; (2) the ability to articulate their thoughts and express themselves clearly; and (3) whether they were working in a rural context.^18^ A variety of HCPs from different disciplines were included in this study in order to ensure that the voices of several members of the healthcare team were heard, many of whom play a significant role in service delivery in rural areas.^19^ In addition, women's experiences may be substantially different from those of men – particularly the experiences of rural women – and it was important that the voices of women should also be heard.

The six graduates who were selected (details provided in Table 1) are referred to throughout the rest of this article by their initials only.

**TABLE 1:** List of those healthcare professionals who participated in the study.

Name	Current position	Professional qualification	Professional experience	Years of professional experience	Age	Gender	Originally from
DG	Student mentor coordinator based in Mtubatuba	BSc Physiotherapy UKZN 2003	2004–2008 Physiotherapist 2008 – current mentor coordinator	11 years	37 years	M	Ingwavuma
FN	Sub-Saharan coordinator Brian Holden Eye Institute	BSc Optometry UKZN 2003	2004–2009 Optometrist – Mosvold, Pelopele train 2010 – current Brian Holden Eye Institute	11 years	34 years	M	Ingwavuma
SM	Psychologist at Hlabisa Hospital	MSc Clinical psychology UJ 2009	Clinical psychologist Hlabisa Hospital 2010 – current	4 years	29 years	M	Ingwavuma
TM	Physiotherapist at Emmaus Hospital	BSc Physiotherapy Wits 2004	Physiotherapist 2005 – current	6 years	33 years	M	Ingwavuma
NM	Pharmacist Military research post in Mtubatuba	BSc Pharmacy Wits 2004	Pharmacist 2005 – current	10 years	35 years	F	Ingwavuma
LH	Medical officer Mseleni Hospital	MBChB UKZN 2006	Intern / community service officer / Medical officer 2007 – current	9 years	30 years	F	Ubombo

F, Female; M, Male; UKZN, University of KwaZulu Natal; UJ, University of Johannesburg; Wits, University of Witwatersrand.

### Data collection

**FIGURE 1:**
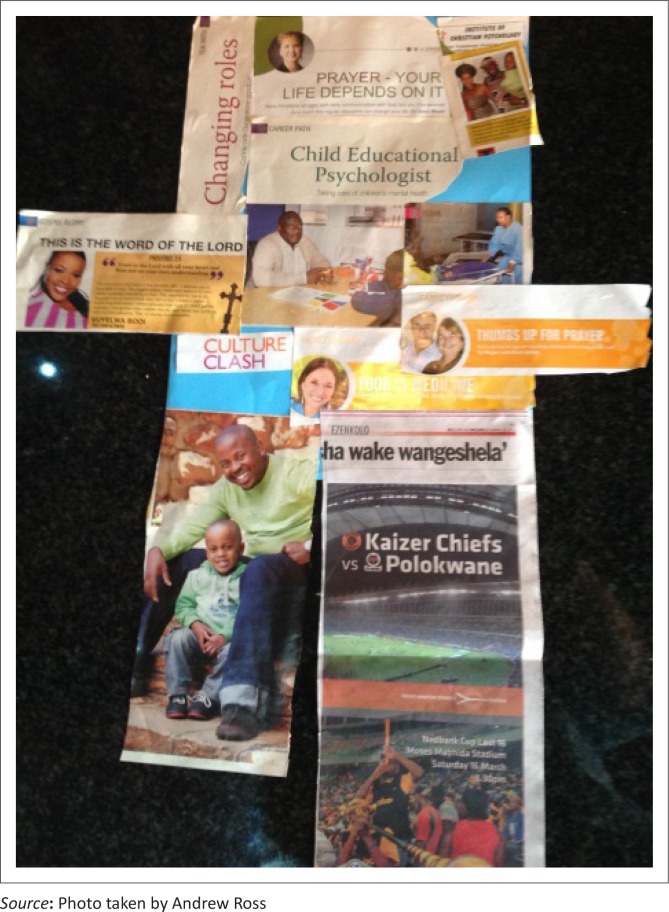
Collage development [SM].

Data were collected by the author using two unstructured interviews where participants were asked the question, ‘tell me about your educational experiences from rural scholar to healthcare professional and what it means to work in a rural setting’. Interviews were supplemented by photographs and artifacts from different stages of their educational experiences and by the construction of a collage of a day in their lives (see Figure 1). Participants were asked to describe the photographs and/or artifacts and/or pictures chosen for the collage and how they were connected to their educational experiences. All interviews and discussions were recorded on a voice recorder and transcribed verbatim.

**BOX 1:** Discussion of collage in Figure 1.

In the discussion following the construction of the collage, SM highlighted the mportance of his job as a clinical psychologist and the important role that he was playing in the community. He also mentioned the contribution that he was making as a clinical psychologist and as a graduate in the community. He also talked about his changing role within his family, the importance of family and the fact that he was looking forward to having a family of his own. The importance of his religious beliefs, along with how these had impacted upon his choice to return to work at the local district hospital, were also discussed, as well as his love for soccer.

### Data analysis

A reconstructed story was written from the transcripts^20^ and was sent to the participants for content validity. The stories were read and re-read, codes and categories identified, patterns and relationships between categories reviewed and themes developed.^18^

### Ethical consideration

Ethical approval was obtained from the Social Science Ethics Committee of the University of KwaZulu Natal (HSS/1205/012D). Written informed consent, including possible identification, was obtained from all of the participants after the aims of the study were explained to them.

## Results

These UYDF SS graduates were chosen to participate in this study because they have continued to work in rural areas. They all had a year-for-year work-back obligation to UYDF to return to the area they came from. Although this was important, because ‘… I wanted to fulfill my contract with Friends of Mosvold’ [DG], fulfilling a contractual obligation was not the primary reason for returning to the hospitals.

SM had a ‘great offer to run an employee wellness programme from one of the mining houses’. He could have bought himself out of the contract and remained in Johannesburg, but because of his personal commitment he chose ‘… to come back for the community. Not returning would have been cheating. I had this moral obligation to my mum and the community’. There were also community expectations that students, who had been chosen by a committee made up of hospital and community members, would return to work on the area after graduating. Graduates were motivated to return because:

‘… [*p*]eople kept on asking me “when are you coming back?”. So that kind of put pressure on me … they must see me coming back now.’ [DG]

Working at the local district hospital enabled some of these graduates to stay at home. Even those who had accommodation at the hospital found it easy to visit and provide support to their families. An additional advantage of living in a rural area was that it was cheaper than staying in an urban area because less money was spent on transport as home was close to work and there were less things to spend money on:

‘… [*M*]oney-wise, you don’t really spend as much as you would be spending in the urban areas. If you really want to save, you can, and that this [*sic*] was an important advantage of working at a rural district hospital.’ [NM]

The following themes were identified from the data:

- Contributing to service delivery.
- Professional development.
- The challenges and frustrations of working in rural hospitals.
- Personal, family and community impact of working as a healthcare professional.

### Theme one: Contributing to service delivery

These professional graduates were able to contribute toward service delivery:

‘… [*W*]e worked hard seeing patients in the wards and in OPD [*the outpatient department*] and visiting the clinics.’ [DG]

Having grown up in rural areas, they understood what life was like for the majority of the population and this influenced their work ethic and attitude to work. SM recognised that:

‘… [*i*]f I don’t go to a certain clinic, there's a person there who is waiting for me, who has only that option, to go to that clinic, and nothing else.’

Graduates were able to extend the services provided:

‘I saw the therapy department grow from one therapy assistant visiting 16 out of the 34 clinics on her own … to four physios, two OTs [*occupational therapists*], two audiologists and two speech therapists.’ [DG]

Using contacts established through UYDF, they were also able to raise money for equipment needed to support these services.

They brought new skills to the hospitals:

‘… I made it my responsibility to make eye services a priority in the whole district … because of my rural origin I am passionate about making sure that quality healthcare is delivered to people.’ [DG]

These graduates were proactive in motivating for posts to be created, staff to be recruited and equipment to be purchased:

‘… [*T*]hings never happen by chance … I always had a plan for the eye clinic.’ [FN]

Meaningful contributions were made both to patient care and to the training and support of other HCPs:

‘… [*W*]hen I started, the TB [*tuberculosis*] cure rate was below 40%; by the end of the first year, we managed to get it up to 60%, then we got it up again to 75%.’ [LH]

‘… I orientate [*sic*] them about the HIV and TB programmes.’ [LH]

### Theme two: Professional development

These HCPs found the work to be interesting, varied and stimulating, with DG commenting that:

‘… I remember seeing conditions that you would never see at university – I used to phone the lecturers and say “I’m seeing this, what is it?” Seeing those kind of conditions helped me to say, “[*L*]et me learn more.”’

They were also able to take advantage of opportunities for professional development. LH was involved in ‘a research project on multiple drug resistant TB’ and is ‘… currently working on modifying the new antiretroviral guidelines’.

SM has had the opportunity to develop professionally as he learnt more about community psychology and culture-bound psychology.

There were also opportunities for promotion which happened more quickly than would have happened in urban hospitals and at some hospitals they felt valued and appreciated by the management, other staff members, patients and members of their community. For most, working in a rural district hospital:

‘… [*h*]as been a wonderful journey and I’m loving what I’m doing … I’m developing caring, supportive relationships and working as a healer and comforter. I see my work as somebody who brings hope to the sick.’ [LH]

### Theme three: Challenges and frustrations

Graduates, however, found that there were significant challenges and frustrations when working at rural district hospitals. These graduates were chosen by a local selection committee which included community members and management based on a prioritisation of services needed. As students they had returned to the hospital for work-based experience every year and management were aware of their graduation date. Despite this:

‘… [*t*]here were no [*optometry*] posts [*when I graduated in December*] … I eventually started work on the 5th of April in an oral hygienist post; there was no job description and the necessary equipment was not available to function [*sic*].’ [FN]

Keeping graduates and making them feel that they were valued and important members of the hospital staff did not appear to be a priority for hospital management at some hospitals. At these hospitals, management was not perceived to be proactive in the creation and filling of posts or in the provision of basic facilities such as suitable accommodation:

‘I was willing to fulfill my five year contract but the hospital could only offer me accommodation in a dorm in the Nurses Home with the student nurses although the hospital offered houses to other professional staff.’ [DG]

Although management had high expectations of them, graduates often did not feel supported or appreciated:

‘… I was left alone to do the job of three [pharmacists] … [*T*]hey told us that we could not close at four but needed to work until five pm but there was no money for overtime. They offered us the opportunity to take time back but it wasn’t easy to take time off when I was the only pharmacist.’ [NM]

All of the graduates experienced many frustrations whilst working at the hospitals. LH remembered the following regarding working as a newly-qualified doctor:

‘… [*D*]uring a particularly difficult and stressful operation … I called one doctor. He told me “I’m not on call today. You are a doctor, just see how you can handle it.”’

She concluded with:

‘… [*T*]hat's how rural medicine is like. Everyone always said rural medicine was horrible and I experienced that first hand.’

She had planned to work at a rural hospital for a number of years, but after that experience she decided:

‘… I couldn’t stay in rural medicine; the only option was just to quit and go do fashion designing. So I waited for the end of the year and in December when my contracted ended I packed everything and said, “I’m leaving medicine” and I stayed at home without a job until I was approached by Dr F who asked me to work at Mseleni hospital.’

Opting out and leaving rural medicine was an understandable consideration for some of these HCPs when working in an unsupportive environment and faced with overwhelming challenges. However, the HCPs who participated in this study remained and continue to work in rural areas. Some of them (LH, DG, NM, TM) moved to other rural, more supportive institutions whilst others found perspective and were able to continue to work in the same hospital (FN, SM). However even when moving to another hospital, perspective was important as there are challenges in every environment:

‘… I realised that there are problems wherever you go … [*I*]f you can make a difference here, you can make a difference anywhere. You just need to find a solution to whatever challenges you are facing.’ [DG].

### Theme four: Personal, family and community impact

For these rural-origin scholars, graduation brought status and respect to them and to their families and has changed their lives forever:

‘Graduating changed me and changed the way people relate to me and my family. Graduation brought a belief that I could do things.’ [FN]

Working as an HCP meant that they had material resources which they used to build houses for their families, provide water and electricity at home and make sure that other members of their family had opportunities to further their education:

‘… Since finishing “varsity”, I have helped my eldest sister. After matric she spent two years at home without doing anything as she couldn’t continue because of the money. I supported her to go college.’ [TM]

These graduates were looked to for health advice as:

‘… I can diagnose and give information to improve their condition.’ [DG]

They are seen as role models in the community, helping to inspire others to undertake their own educational journey:

‘When I speak at schools I start off telling them where I come from and a lot of them get surprised … [*W*]hen I tell them, “this is what has happened to me – you can make it”, that's when they start relating to the message. I start at the beginning and then go on to tell them about the options.’ [SM]

However, perhaps even more profound than being a role model to scholars in the area, was the impact they had on the wider community. Returning to work in the areas and sharing their educational experience with others has changed the communities’ perspective regarding the value of higher education:

‘… [*T*]hrough seeing us, through us talking to them they then got encouraged and now maybe 20% of people in that rural area are taking their kids to places of higher learning. When we started, no-one was sending their kids to university. But now, people in the community sell their cows to send their kids to university because they are able to see what education can do for other kids. That's why quite a lot of kids have gone to university from that side – not only kids from good families, but from poor families as well. Parents are putting in their last pennies for their kids to go to university because they can see it does change the family structure, the economics and all that. It's massive. From what I see now. It's massive.’ [FN]

## Discussion

Using a life history methodology enabled these rural-origin HCPs to tell their life story within the context in which they worked. The stories provided a window to understand the meanings that they attached to their experiences and how they made sense of their world as they worked as an HCP at a rural hospital. Interviews tend to be linear and limited by words whilst the use of arts-based techniques allowed these HCPs to move away from linear thought. Photographs, artifacts and pictures selected for the collages have meaning and memory associated with the experience that the photograph, artifact or picture represents. Using these techniques broadened and deepened my understanding of their experiences as they recalled experiences and explained the meaning relative to the photo or the artifact or the pictures chosen.^21^ The use of these tools in this research project was innovative and provided insight and a better understanding of their experiences of working as an HCP in a rural context.^22,23^

A number of recruitment and retention strategies have been proposed with regard to staffing rural hospitals. These include: educational interventions (rural recruitment, early exposure to rural sites, rural campuses); coercion (compulsory service, regulatory requirements); incentives (increased pay, more holidays); and the creation of an enabling environment (increased support, community appreciation). The UYDF SS, as a rurally-based scholarship scheme, could be considered both a recruitment tool in getting graduates to rural areas and a retention tool in the year-for-year work-back obligation imposed on the graduates. The success of the scheme in encouraging graduates to return and fulfil their work-back obligations supports the evidence from international observational studies that the recruitment and training of rural-origin students is an effective strategy for the long-term staffing of rural facilities.^7,24^ Studies from Australia have shown that that rural origin students are twice as likely to work in rural areas than graduates from urban areas.^25^ Studies from Canada suggest that rural doctors are five times more likely to have originated from rural areas than from urban areas.^26^

In keeping with other studies, reasons given by these graduates for choosing to return to work in a rural district hospital included being able to make a contribution to their communities and to their families, as well as the opportunity to live close to home.^27,28^ It was interesting to note that it was not predominately their contractual obligation which motivated them to return but rather a personal commitment to themselves and their community.

A World Health Organization report published in 2013 reiterated the critical role that HCWs play in improving healthcare, stating that governments must prioritise the training, recruitment and retention of HCWs as there can be ‘no health without a workforce’.^29^ Having and retaining these rural origin HCPs at a district hospital meant that health services could be provided and extended and important services such as optometry and psychology could be introduced. The graduates’ intimate knowledge of the community fueled their passion to both improve and extend the healthcare service. Having graduates who understand the local conditions and who appreciate the value and the need for their services is an important asset which should contribute toward the provision of a service of excellence. These graduates found their work to be both interesting and challenging. Opportunities were provided for professional development, they had opportunities for promotion and they felt valued and appreciated by the community. These experiences are similar to the reasons given for remaining at a district hospital by community service officers who were already working in rural district hospitals in South Africa. HCPs who feel valued and appreciated by management, their patients and community members and who feel that they are providing a worthwhile service have been shown to be more likely to choose to remain in a rural setting.^27^ Purohit has identified these as being the content factors and/or intrinsic motivators which contribute to HCW motivation, quality of work, job satisfaction and a willingness to remain in rural areas.^30^

However, whilst working at rural district hospitals, many of these graduates experienced multiple frustrations and challenges – often with regard to issues such as a lack of posts, lack of policies (no job description), lack of equipment, inadequate accommodation and poor supervision. Purohit has identified these as context or hygiene factors, which are things that should be in place for the smooth running of a healthcare institution. If they are absent, they can lead to high levels of frustration and dissatisfaction. Context and content factors are complementary and not opposites and both need to be addressed if HCPs are to be retained at rural district hospitals.^30^ For example, dealing with accommodation issues may reduce frustration but does not necessarily increase motivation. Doing interesting and worthwhile work which is valued by the community may be motivating but could be undermined by high levels of frustration and plans to leave if, for example, salaries are not paid on time. In this study, four of the six graduates (DG, LH, NM, DG) moved from one district hospital to another, mainly because of context factors. If management wants motivated and productive HCPs who chose to stay and work in rural areas, attention must be given to addressing context factors, in order to reduce and/or eliminate frustrations, as well as to content factors that both motivate and provide a sense of purpose because of doing something that is worthwhile.

The graduates who participated in this study are contributing to service delivery and, in turn, are having a profound influence on their families and on their communities. However the main long-term impact that these graduates have may not be on the health services that they provide but rather on their ability to inspire others to study and to see the potential in education. Ray (2006) has commented that, in relation to aspirations to achieve a better life for oneself and one's family, ‘there is no experience quite as compelling as the experience of your immediate family and more broadly those in your socioeconomic and spatial neighbourhood’.^31^ The experiences of these rural-origin HCPs echo the words of Nelson Mandela:^32^

Education is the great engine of personal development. It is through education that the daughter of a peasant can become a doctor, that the son of a mineworker can become the head of the mine, that a child of farm workers can become the president of a great nation …

### Strengths and limitations

The strength of this study lies in the fact that it gives voice to rural-origin HCPs, enabling them to share their experiences. The small number of participants and the qualitative nature of the study mean that the findings cannot be generalised to other settings. However, it is hoped that those reading this study will be able to identify with the participants and apply what has been learnt to their own context.

## Conclusion

This study has shown that the recruitment and training of rural scholars is a worthwhile, viable, long-term strategy for the staffing of rural institutions in a developing country such as South Africa and that a scholarship scheme can be a successful strategy for both recruitment and retention. These graduates found their work to be both satisfying and enjoyable and were able to provide and extend healthcare services. They gained status and respect within the community and were role models to scholars in the area. Access to resources improved conditions at home and changed the trajectory of the lives of their family members. However, if such a scheme is to be an effective long-term strategy for the recruitment and retention of HCPs for other rural areas, managers needs to invest in the effort of finding and supporting such rural origin scholars. They also need to give attention to addressing context factors (which lead to frustration) and content factors (that promote motivation) in the workplace.
